# Development of an Equine Groove Model to Induce Metacarpophalangeal Osteoarthritis: A Pilot Study on 6 Horses

**DOI:** 10.1371/journal.pone.0115089

**Published:** 2015-02-13

**Authors:** Ugo Maninchedda, Olivier M. Lepage, Monika Gangl, Sandrine Hilairet, Bernard Remandet, Francoise Meot, Geraldine Penarier, Emilie Segard, Pierre Cortez, Christian Jorgensen, Régis Steinberg

**Affiliations:** 1 Equine Research Centre, University of Lyon, VetAgro Sup, Veterinary Campus of Lyon, GREMERES-ICE, Marcy l’Etoile, France; 2 Exploratory Unit, Sanofi-aventis Recherche, Montpellier, France; 3 Clinical unit for osteoarticular diseases, CHU Lapeyronie University Hospital, Montpellier, France; Instituto Butantan, BRAZIL

## Abstract

The aim of this work was to develop an equine metacarpophalangeal joint model that induces osteoarthritis that is not primarily mediated by instability or inflammation. The study involved six Standardbred horses. Standardized cartilage surface damage or “grooves” were created arthroscopically on the distal dorsal aspect of the lateral and medial metacarpal condyles of a randomly chosen limb. The contralateral limb was sham operated. After 2 weeks of stall rest, horses were trotted 30 minutes every other day for 8 weeks, then evaluated for lameness and radiographed. Synovial fluid was analyzed for cytology and biomarkers. At 10 weeks post-surgery, horses were euthanized for macroscopic and histologic joint evaluation. Arthroscopic grooving allowed precise and identical damage to the cartilage of all animals. Under the controlled exercise regime, this osteoarthritis groove model displayed significant radiographic, macroscopic, and microscopic degenerative and reactive changes. Histology demonstrated consistent surgically induced grooves limited to non-calcified cartilage and accompanied by secondary adjacent cartilage lesions, chondrocyte necrosis, chondrocyte clusters, cartilage matrix softening, fissuring, mild subchondral bone inflammation, edema, and osteoblastic margination. Synovial fluid biochemistry and cytology demonstrated significantly elevated total protein without an increase in prostaglandin E2, neutrophils, or chondrocytes. This equine metacarpophalangeal groove model demonstrated that standardized non-calcified cartilage damage accompanied by exercise triggered altered osteochondral morphology and cartilage degeneration with minimal or inefficient repair and little inflammatory response. This model, if validated, would allow for assessment of disease processes and the effects of therapy.

## Introduction

Osteoarthritis (OA) in humans and horses [1] is characterized by progressive and permanent deterioration of articular cartilage combined with changes in the subchondral bone and soft tissues of the joint [2–5]. OA can occur in young athletes [6] or older individuals [5], and its prevalence and similarities between horses and humans [1] has led to the use of equine OA models.

In horses, OA frequently affects the metacarpophalangeal (MP) joint [6, 7]. No disease model has produced satisfactory degenerative changes in the entire joint to enable assessment of disease processes or measurement of the effects of new therapies without the confounding of instability or inflammation. For example, impact injury of the palmar aspect of the metacarpus (MC3) causes only mild focal OA [8] while overload [9–11] and cast immobilization [12, 13] of the MP joint create degenerative changes that are too limited and inconsistent. Transection of the lateral collateral ligament and lateral collateral sesamoidean ligaments of the MP joint induces degenerative changes with permanent instability [7], and osteochondral (OC) fragment-plus-exercise models in the MP joint [14] or the mid-carpal joint [15–18] produce post-traumatic OA accompanied by significant synovial inflammation.

A traumatic model of joint degeneration not primarily mediated by instability or joint inflammation, known as the groove model, has been described for the canine stifle joint [19–21] and the ovine MP joint [22]. In these models, a surgically applied injury to the articular cartilage is created by scratching the surface on the weight-bearing area. The purpose of our study was to develop an equine MP joint groove model. We hypothesized that a standardized arthroscopic grooving procedure with controlled exercise would induce MP joint OA. Horses were evaluated for lameness, radiological signs, and synovial fluid cytology and biomarkers. At 10 weeks post-surgery, horses were euthanized for macroscopic and histologic joint assessment.

## Materials and Methods

### Animals

Six French Standardbreds, owned by the Lyon equine research group (GREMRES), were included. The group comprised five geldings and one mare with a mean age of 6.2 ± 0.5 years and a mean weight of 543 ± 8 kg, without forelimb lameness. The Ethical Committee of VetAgro Sup approved the protocol (Permit Number: 69 127 800, Certified Number: B 69 127 0501) (S1 ARRIVE Guidelines Checklist). All surgery was performed under isoflurane anesthesia, and all efforts were made to minimize suffering.

### Surgery and Exercise Program

Before surgery, the groove MP joint was randomly chosen while the contralateral joint was used for the sham procedure. Arthroscopic surgery was performed under general anesthesia in dorsal recumbency with both anterior MP joints prepared and draped for aseptic surgery. The arthroscope was inserted through a dorsolateral portal made lateral to the common digital extensor tendon.

Before the grooving procedure was begun, the dorsal aspects of the MP joints were arthroscopically assessed for the presence of pre-existing degenerative lesions on the dorsal aspect of MC3 condyles (wear lines, erosions, fragments, fissures, chondromalacia). Lesions were documented using high-resolution digital video and photographs.

**Figure 1:**
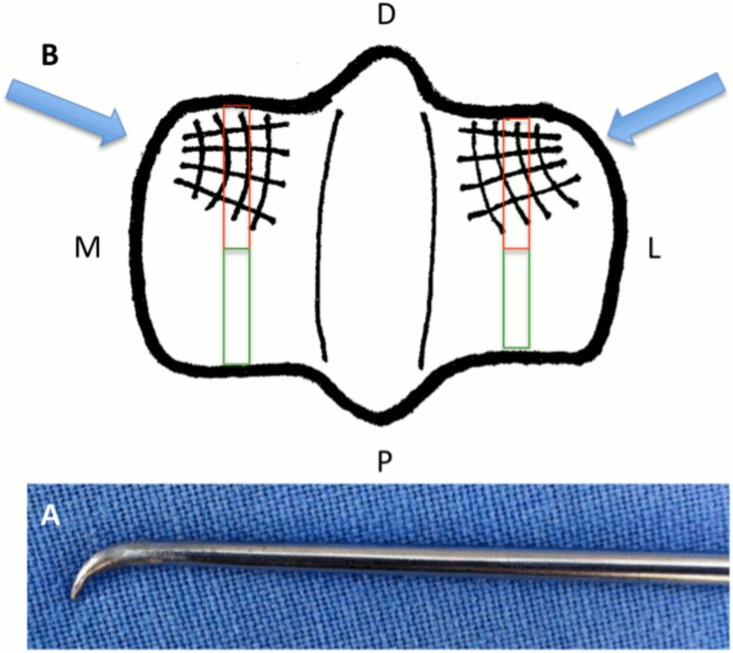
(A) Modified arthroscopic probe in which the tip was hooked and sharpened to a 2 mm internal length. (B) The distal MC3: D = dorsal, P = palmar, L = lateral, and M = medial. A woven pattern of 4 dorso-palmar and 4 radial grooves created on the weight-bearing area of the lateral and medial condyles. Blue arrows represent the direction the instrument was introduced. Red and green rectangles represent the dorsal histologic slices centered over the groove areas and the palmar histologic slices in the continuity of the dorsal slices.

Under maximum joint flexion, an 18 G needle was used to ascertain the ideal position of the instrument portal, as laterally and medially as possible. Cartilage of the most distal and dorsal aspects of the medial and lateral MC3 condyles was grooved with a customized modified arthroscopic probe in which the hooked tip was sharpened to a 2 mm internal length (Fig. 1A). A woven pattern made of four dorso-palmar and four radial grooves was created on the weight-bearing area of both condyles (Fig. 1B). Grooving was performed under arthroscopic guidance with gas (CO_2_) distension followed by 3 minutes of lavage with lactated Ringer’s solution.

Skin incisions were sutured with 2/0 polypropylene monofilament (Premilene B-Braun), and surgical sites were protected with sterile bandages. During surgery, each horse received an anti-inflammatory (intravenous phenylbutazone 2.2 mg/kg every 12 h) and an antimicrobial (intramuscular penicillin procaine 22,000 UI/kg every 12 h). These medications were continued for 24 hours in the hospital as routine post-arthroscopic medications. Bandages were changed after 24 hours and then every 3 days until suture removal at 12 days post-surgery.

Post-operatively, horses were stall rested for 2 weeks. For the following 8 weeks, they were turned out in a common 2,000 m^2^ paddock while on an exercise program that consisted of trotting in a 10-m circle on a sand surface every other day for 15 minutes in each direction.

### Radiographic Analysis

Six radiographic projections of both anterior MP joints including lateromedial, dorsopalmar, flexed dorsopalmar, flexed lateromedial, 45-degree dorsolateral-palmaromedial, and dorsomedial-palmarolateral obliques were obtained at weeks 0 and 10 (Fuji CR, Fujifilm Medical Systems). The severity of radiographic changes was semi-quantitatively graded from 0 to 2 (0: normal; 1: mild to moderate; 2: severe) for synovial effusion, osteophytes at 12 sites (dorsal, palmar, lateral, and medial MC3 condyles; dorso-lateral, dorso-medial, palmaro-lateral, and palmaro-medial first phalanx (P1); and latero-proximal, latero-distal, medio-proximal, and medio-distal proximal sesamoid bone (PSB)), subchondral bone sclerosis, and subchondral bone lysis at 7 sites (medial and lateral MC3 condyle, medial and lateral P1, sagittal groove, medial and lateral PSB). The joint space width (JSW) was measured on the middle surfaces of the lateral and medial condyles on dorsopalmar view and semi-quantitatively graded for inclusion in the total radiographic score. All radiographs were blindly evaluated by one board-certified veterinary pathologist (ES). The total radiographic score (0–56) consisted of synovial effusion (0–2), osteophytes (0–24), subchondral bone sclerosis (0–14), subchondral bone lysis (0–14), and JSW (0–2).

### Lameness Evaluation

Before the grooving procedure (at baseline) and at 2-week intervals, all horses underwent a clinical lameness examination, including distal forelimb flexion tests. The American Association of Equine Practitioners’ lameness scale was used (0 = normal; 1 = lameness difficult to observe and not consistently apparent regardless of circumstances; 2 = lameness difficult to observe at a walk or trotting a straight line but consistently apparent under certain circumstances; 3 = lameness consistently observable at a trot under all circumstances; 4 = obvious lameness at a walk; 5 = minimal weight bearing) [23]. Objective lameness examinations were performed using an inertial sensor-based system (Lameness Locator, Equinosis, Columbia, MO, USA). Lameness was measured using the A1/A2 ratio to represent asymmetry in the right or left forelimb, and the vector sum was calculated to represent forelimb lameness [24, 25]. Clinical and objective lameness evaluations were performed by one individual (UM) who was aware of the treatment.

### Synovial Fluid

Synovial fluid samples were collected in EDTA tubes during surgery and on days 3, 7, 30, and 70. Samples were centrifuged at 1500 ×*g* for 10 minutes at 4°C. The supernatants were stored at -20°C pending biomarker analyses while the precipitates were evaluated for leukocytes.

Total protein (TP) concentration was measured using a commercial kit (TP Gen.2, Roche Diagnostics). The following biomarkers were assayed with various commercial ELISA kits: PGE_2_ (R&D Systems, Minneapolis, MN, USA) with indomethacin additive to measure inflammation; C2C collagen type II cleavage (IBEX Diagnostics, Montreal, Quebec, Canada) to measure collagen II turnover; COMP (Euro-Diagnostic, Malmo, Sweden) to measure cartilage metabolism; CS-846 or aggrecan with hyaluronidase additive and carboxypeptide of CPII (IBEX Diagnostics, Montreal, Quebec, Canada) to measure cartilage synthesis; CTX-I (Immunodiagnostic Systems, Boldon, UK) to measure bone resorption; and osteocalcin (Quidel Corporation, San Diego, CA, USA) to measure bone formation [26].

Samples of synovial fluid were stained with Wright’s stain for cytological examination. Smears were microscopically examined and semi-quantitatively scored from 0 to 6 (0: absence; to 6 very many) for neutrophils, synoviocytes, and chondrocytes (including clusters). Cytological evaluations were blindly evaluated by one board-certified veterinary pathologist (BR).

### Postmortem Examination

At 10 weeks post-surgery, all horses were submitted to euthanasia with an intravenous injection of a mixture of 1 g embutramide, 2.5 g mebezonium, and 250 mg tetracaine hydrochloride (T-61, Intervet, Kirkland, Quebec, Canada).

**Macroscopic Examination and Scoring**. Both anterior MP joints were opened from the dorsal aspect, exposing the joint surfaces. Wear line, erosion, and palmar arthrosis changes on the MC3 condyles were scored according to the semi-quantitative macroscopic guidelines of the Osteoarthritis Research Society International (OARSI) [27]. Similarly, wear line and erosion on P1 and PSBs were scored. A total MC3 macroscopic score of 0 to 21 consisted of a MC3 wear line score (0–3), MC3 erosion score (0–3), MC3 palmar arthrosis lesion score (0–3), P1 wear line score (0–3), P1 erosion score (0–3), PSB wear line score (0–3), and PSB erosion score (0–3). Synovial membrane hypertrophy and inflammation were also scored, each from 0 to 3. The grooved areas were excluded from this grading scheme and were independently evaluated histologically. Macroscopic evaluations were blindly performed by two individuals (BR, a board-certified pathologist, and UM).

**Microscopic Examination and Scoring**. The distal end of the MC3 and the synovial membrane from the dorsal pouch of each anterior MP joint were sampled and fixed with neutral buffered formalin (Carlo Erba Reagents). After fixation, slices 3–4 mm thick of the dorsal and palmar aspects were taken of the middle part of the medial and lateral MC3 condyles. The dorsal slices were centered over the grooved areas to allow assessment of microscopic changes surrounding the grooves, and the palmar slices were in the continuity of the dorsal slice for assessment of microscopic changes away from the grooves (Fig. 1). These slices were decalcified using DC3 decalcifying solution (Labonord SAS, Templemars, France). The decalcified condyle slices as well as 3–4-mm slices of the synovial membranes were processed on histological slides. The condyle slides were stained with hematoxylin and eosin, Safranin O, and Alcian blue while the synovial slides were stained with only hematoxylin and eosin.

Osteochondral damage on each dorsal and palmar MC3 was evaluated using modified OARSI histological guidelines [27]. Each feature of the non-calcified cartilage, calcified cartilage, and subchondral bone was scored from 0 (no change) to 4 (marked change). Scored features of the non-calcified cartilage were chondrocyte necrosis (i.e., chondrocyte-free areas or empty chondrocyte lacunae, or focal cell loss), chondrocyte clusters (i.e., complex chondrone formation), fibrillation or fissuring, cartilage matrix softening (i.e., cartilage matrix appearing more or less pale and loose, visible in all stains), vertical grooves (i.e., surgically induced grooves crossing the entire thickness of the non-calcified cartilage extending to the junction with calcified cartilage), horizontal cracking (i.e., cracks at the junction of the non-calcified and calcified cartilages), and detachment of non-calcified cartilage. The calcified cartilage was scored with respect to chondrocyte necrosis and fissuring. The subchondral bone was scored with respect to fissuring, osteoblastic margination (i.e., basophilic osteoblastic cells aligned at the border of subchondral bone cavities), edema, congestion, and subacute inflammation. A total cartilage score (0–32) was determined by adding the overall non-calcified cartilage score at the exclusion of vertical grooves (0–24) and the overall calcified cartilage score (0–8), while a total subchondral bone score (0–20) was determined separately. Scores were compared between groove and sham joints and between dorsal and palmar slices of each joint.

The synovial membrane was evaluated for thickening of the intimal layer (with or without inflammation), perivascular inflammation in the sub-intimal layer (with or without fibrosis), and synovial folds. Microscopic assessment was blindly performed by one board-certified veterinary pathologist (BR).

### Statistical Analysis

The global normality test for A1/A2 yielded *P* = 0.357, suggesting normal distribution. The difference in A1/A2 lameness values between sham and groove limbs was determined using a one-way ANOVA followed by the Dunnett test. Biochemical values were normally distributed, and their variances were equal, as revealed by the Levene test. Biochemical data were analyzed with a two-way repeated-measures ANOVA (post hoc test using winner). Cytology scores were not normally distributed and were analyzed with non-parametric two-way repeated-measures ANOVA. Radiographic, macroscopic, cytologic and histologic scores were represented by the median ± median absolute deviation. Radiographic data were analyzed using the Kruskal—Wallis test with a Wilcoxon pairwise comparison. Macroscopic and microscopic scores were analyzed with a Wilcoxon signed-rank test. Microscopic scores difference were analyzed for sham vs groove joints and dorsal vs palmar. A value of *P* < 0.05 was considered to indicate statistical significance. Statistical analysis was performed using a software package recommended by the US Food and Drug Administration (EverStat V6 under SAS v9.2).

## Results

### Arthroscopic Evaluation

At week 0, arthroscopic evaluation showed only limited degenerative changes of the dorsal aspect of MC3. One groove joint had four partial-thickness wear lines on the dorso-proximal aspect of the lateral condyle, and another groove joint had a slight 2-mm fibrillation surface area on the dorso-proximal aspect of the medial condyle.

### Lameness Evaluation

**Figure 2:**
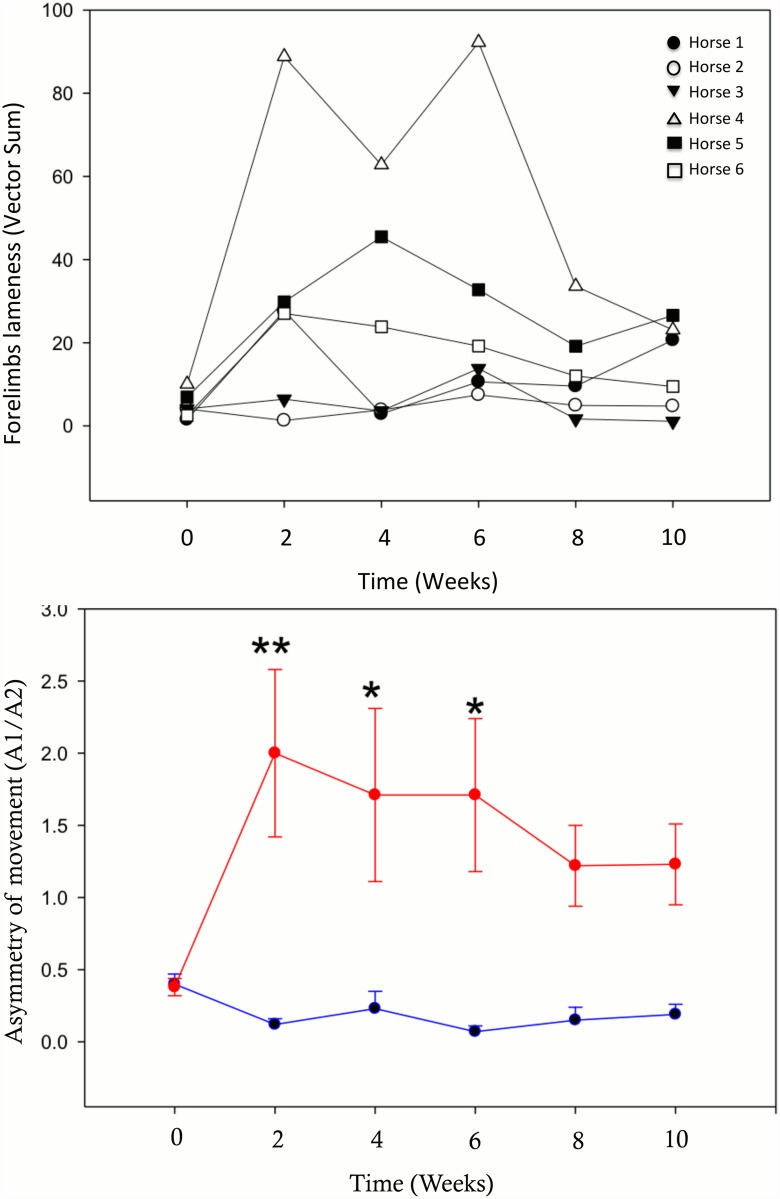
Lameness evaluation with the inertial sensor-based system. (A) Asymmetry of movement of the groove limb (red) as opposed to the sham limb (blue), measured by A1/A2. Mean ± SEM is shown. Asterisks represent significant differences between the groove and sham limbs (*P < 0.05, **P < 0.005). (B) Individual time-course of lameness measured by vector sum.

At 2 weeks post-surgery, all horses were consistently grade 2–3 lame with a positive distal limb flexion test on the groove forelimbs. Measured mean A1/A2 values showed significant asymmetry in the movement of the groove forelimbs during the first 6 weeks post-surgery. This asymmetry decreased but remained present through weeks 8 and 10, but the difference between groove and sham limb became statistically non-significant (Fig. 2A). Vector sums demonstrated individual variability of the overall measure of forelimb lameness (Fig. 2B).

### Radiographic Examination

**Figure 3:**
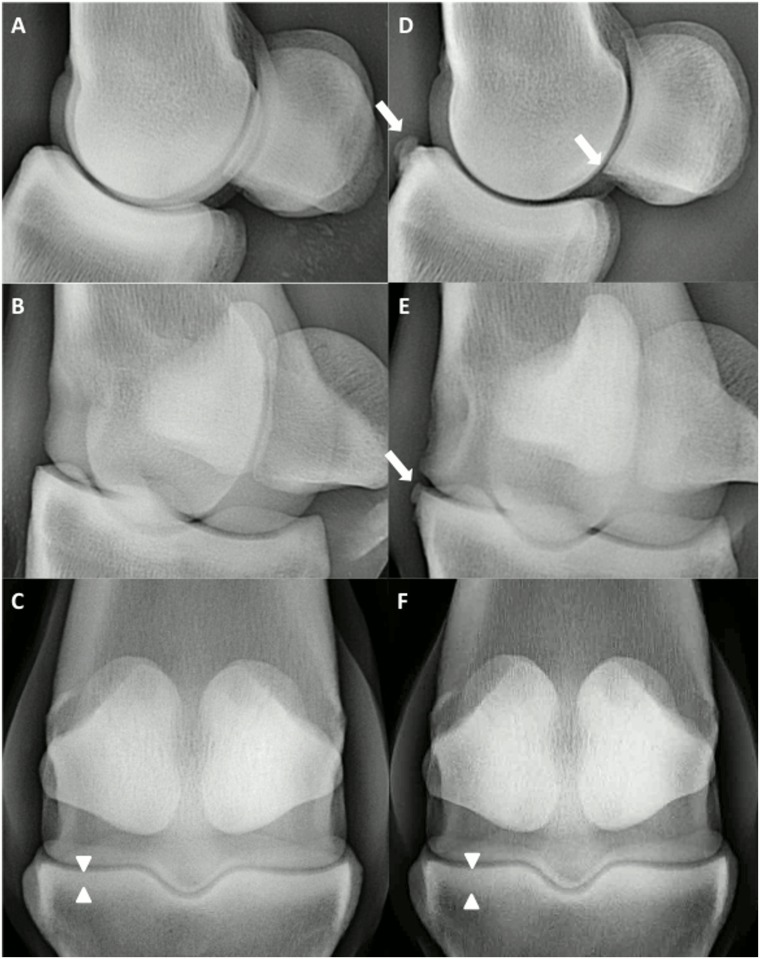
Radiographic views of the MP joint of an individual at week 0 (A, B, C) and week 10 (D, E, F). A and D show the latero-medial view, B and E show the 45-degree oblique view, and C and F show the dorso-palmar view. Grade 1 to 2 osteophytes (white arrows) and grade 1 sclerosis (white triangles) are visible.

The median total radiographic scores of the groove joints at week 10 were significantly higher than at baseline and the sham joints at week 10 (Table 1). The difference was due to significant synovial effusion and osteophytes (Fig. 3).

**Table 1:** Median radiographic scores (± median absolute deviation) for groove and sham joints at weeks 0 and 10.

	**Week 0**		**Week 10**	
	**Groove (n = 6)**	**Sham (n = 6)**	**Groove (n = 6)**	**Sham (n = 6)**
**Radiographic scores**				
**Synovial effusion (0–2)**	0 (±1)	0 (±1)	2 (±2) * ^†^	1 (±1) ^‡^
**Osteophytes (0–24)**	2.5 (±4)	2.5 (±6)	7 (±13) *	1.5 (±6)
**Subchondral bone sclerosis (0–14)**	2.5 (±6)	1 (±3)	2.5 (±3)	2.5 (±5)
**Subchondral bone lysis (0–14)**	0	0	0	0 (±1)
**Joint space (0–2)**	0 (±1)	0	0 (±2)	0 (±1)
**Total radiographic score (0–56)**	4 (±10)	3.5 (±9)	10.5 (±18) *	5 (±12)

* Values represent significant differences between groove joints at weeks 0 and 10 (*P* < 0.05).

^†^ Significant differences between groove joints and sham joints at week 10 (*P* < 0.05)

^‡^ Significant differences between sham joints at weeks 0 and 10 (*P* < 0.05)

### Synovial Fluid

On day 30, TP was significantly higher in the synovial fluid of groove joints compared to sham joints (23.3 ± 5.4 g/L vs 7.7 ±1.5 g/L), then returned to baseline at day 70. PGE2, C2C, COMP, CS-846, CPII, osteocalcin, and CTX-I were not significantly different between groups.

Cytological evaluation revealed that synovial fluid chondrocytes (including few clusters) were absent in both groups until day 70 where chondrocytes were significantly higher in the groove joints compared to sham joints (0.5 ± 0.5 vs 0 ± 0). Neutrophiles and synoviocytes were present with a grade between 1 and 3 but differences between groups were not significant.

### Postmortem Examination

**Figure 4:**
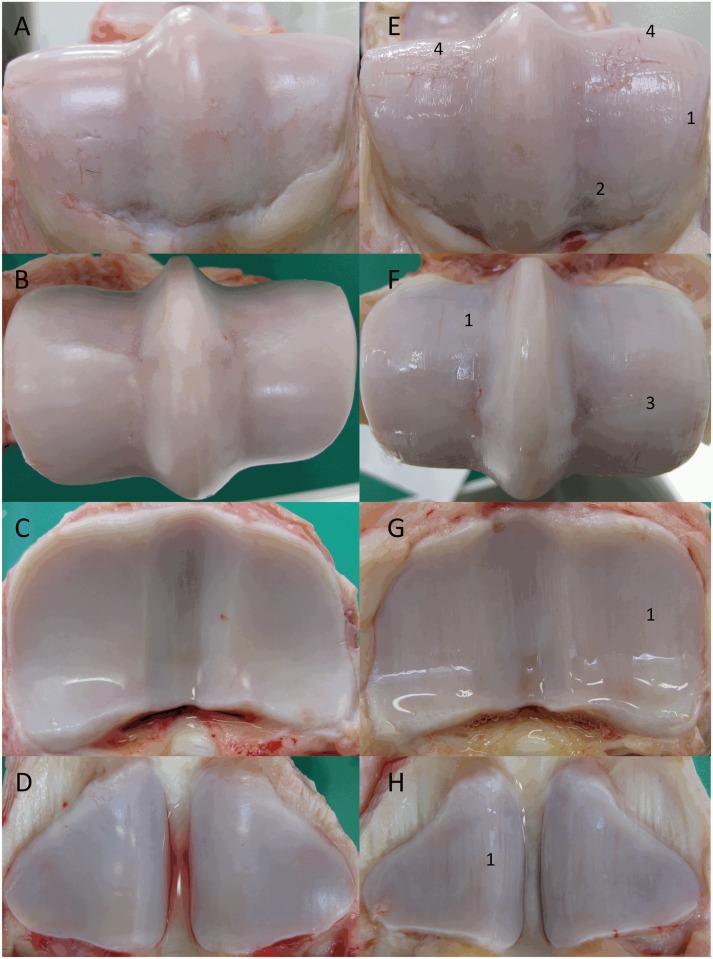
Macroscopic view of the articular cartilage surfaces at week 10 of the sham joints (left) and groove joints (right). (A), (E) are the dorsal aspects of MC3; (B), (F) are the palmar aspects of MC3; (C), (G) are P1; and (D), (H) are PSB. Characteristic grade 2 wear lines (1), grade 3 erosions (2), grade 3 palmar arthrosis (OC lesions of the distal palmar aspect of MC3) (3), and surgical grooves (4).

**Macroscopic scoring**. All sham joints remained normal, demonstrating smooth and white cartilage, while all groove joints had a more translucent and pale appearance. All surgical grooves were similar to one another without signs of healing or fibrin deposition. The median total macroscopic score (excluding surgically grooved areas) was significantly higher among groove joints compared to sham joints (Table 2). Cartilage wear line lesions with bone remodeling on the margins were extended throughout the MP joint, including the P1, MC3, and PSB (Fig. 4).

**Table 2:** Median macroscopic and microscopic scores (± median absolute deviation) for groove and sham joints at week 10.

	**Week 10**	
	**Groove (n = 6)**	**Sham (n = 6)**
**Macroscopic scores (MC3)**		
Wear lines (0–3)	1.5 (±1)	0 (±0)
Erosion (0–3)	1.5 (±0.5)	1 (±0)
Palmar arthrosis (0–3)	2 (±0)	0.5 (±0.5)
**Macroscopic scores (P1)**		
Wear lines (0–3)	2 (±0)*	0 (±0)
Erosion (0–3)	3 (±0)*	1 (±1)
**Macroscopic scores (PSB)**		
Wear lines (0–3)	0 (±0)	0 (±0)
Erosion (0–3)	0 (±0)	0 (±0)
**Total macroscopic score (0–21)**	10 (±1.5)*	2.5 (±2)
**Microscopic score (dorsal MC3)**		
**Non-calcified cartilage**		
Chondrocyte necrosis (0–4)	2.8 (±0.3)* ^†^	0 (±0)
Chondrocyte clusters (0–4)	3 (±0)* ^†^	0 (±0)
Fibrillation/fissuring (0–4)	0 (±0)	0 (±0)
Cartilage matrix softening (0–4)	2.8 (±0.3)*	0 (±0)
Vertical grooves (0–4)	3 (±0)* ^†^	0 (±0)
Horizontal cracking (0–4)	2.8 (±0.3)	0 (±0)
Detachment of non-calcified cartilage (0–4)	1 (±0.5)	0 (±0)
**Calcified cartilage**		
Chondrocyte necrosis (0–4)	2 (±0)* ^†^	0 (±0)
Fissuring (0–4)	2 (±0.5)* ^†^	0 (±0)
**Total cartilage score (0–32)**	15.8 (±2.8)* ^†^	0 (±0)
**Subchondral bone (dorsal MC3)**		
Fissuring (0–4)	0.3 (±0.3)	0 (±0)
Osteoblastic margination (0–4)	2.25 (±0.5)*	0.25 (±0.25)
Edema (0–4)	2.5 (±0.5)	0.25 (±0.25)
Congestion (0–4)	0.5 (±0.5)	0 (±0)
Subacute inflammation (0–4)	0.5 (±0)	0 (±0)
**Total subchondral bone score (0–20)**	6.5 (±1.3)* ^†^	0.5 (±0.5)
**Microscopic score (palmar MC3)**		
**Non-calcified cartilage**		
Chondrocyte necrosis (0–4)	0 (±0)	0 (±0)
Chondrocyte clusters (0–4)	0.5 (±0.5)	0 (±0)
Fibrillation/fissuring (0–4)	0 (±0)	0 (±0)
Cartilage matrix softening (0–4)	0.5 (±0.5)	0 (±0)
Vertical grooves (0–4)	0 (±0)	0 (±0)
Horizontal cracking (0–4)	0 (±0)	0 (±0)
Detachment of non-calcified cartilage (0–4)	0 (±0)	0 (±0)
**Calcified cartilage**		
Chondrocyte necrosis (0–4)	0 (±0)	0 (±0)
Fissuring (0–4)	0 (±0)	0 (±0)
**Total cartilage score (0–32)**	2.5 (±1.75)	0 (±0)
**Subchondral bone (palmar MC3)**		
Fissuring (0–4)	0 (±0)	0 (±0)
Osteoblastic margination (0–4)	0.75 (±0.5)	0 (±0)
Edema (0–4)	2 (±0.75)	0 (±0)
Congestion (0–4)	1 (±1)	0 (±0)
Subacute inflammation (0–4)	0 (±0)	0 (±0)
**Total subchondral bone score (0–20)**	3.8 (±1.8)	0 (±0)

* Significant difference between groove joints and sham joints (*P* < 0.05).

^†^ Significant differences between dorsal and palmar for the groove joints and sham joints (*P* < 0.05).

Chondrocyte necrosis = chondrocyte-free areas or empty chondrocyte lacunae, or focal cell loss.

Chondrocyte clusters = complex chondrone formation.

Cartilage matrix softening = cartilage matrix appeared more or less pale and loose, visible in all stains used.

Osteoblastic margination = basophilic osteoblastic cells aligned at the border of subchondral bone cavities.

Vertical grooves = surgically induced grooves crossing the entire thickness of the non-calcified cartilage extending to the junction with calcified cartilage.

Horizontal cracking = cracks at the junction of the non-calcified and calcified cartilage.

**Cartilage histology**. The median total microscopic cartilage scores for the dorsal aspects of the MC3 condyles were significantly higher among groove joints compared to sham joints. Significant differences were seen in the scores for chondrocyte necrosis, chondrocyte clusters, and matrix softening (non-calcified cartilage characteristics); chondrocyte necrosis and cartilage fissuring (calcified cartilage characteristics); and osteoblastic margination (subchondral bone characteristic) (Table 2). Complex chondrone formation was seen among chondrocyte clusters, and a pale and loose appearance signaled matrix softening. Differences in all other aspects were statistically insignificant. Cartilage vertical grooving, horizontal cracking, and detachment of non-calcified cartilage were worse on the lateral than the medial condyles but not statistically significant.

**Figure 5:**
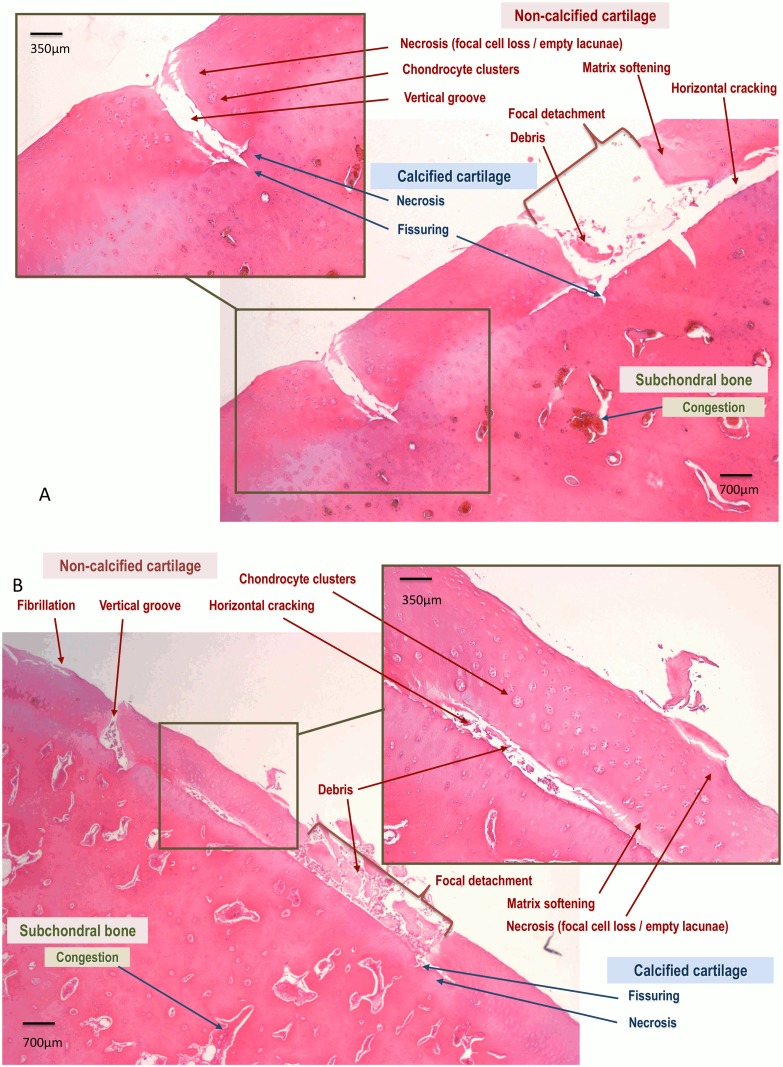
(A and B) Representative light micrographs of the dorsal MC3 condylar cartilage (hematoxylin and eosin stained) obtained from a groove joint at week 10 showing lesions of the non-calcified cartilage (red), calcified cartilage (blue), and subchondral bone (green).

In the grooved areas, horizontal cracking occurred frequently at the non-calcified—calcified cartilage junction, leaving more or less large areas of bare calcified cartilage at the bottom of the vertical grooves and horizontally cracked areas. Focal detachments of non-calcified cartilage also were present, and cartilage fragments were frequently noted in the empty spaces (Fig. 5).

Scores for the palmar aspects of the MC3 condyles between groove and sham joints were not significantly different for any characteristic of the three categories (Table 2). Differences between the dorsal and palmar aspects of the MC3 condyles were significantly different only for the groove joints, for chondrocytes necrosis and chondrocytes clusters scores in the non-calcified cartilage, for chondrocytes necrosis and fissuring scores in the calcified cartilage, for the total cartilage score and for the total subchondral bone score.

In addition, no differences were observed for the synovial membranes between sham and groove joints except for a mild increase in sub-intimal layer thickness in groove joints.

## Discussion

This groove model of the equine MP joint demonstrated that a single traumatic insult to the MC3 cartilage surface accompanied by exercise during the ensuing 10 weeks triggers radiographic, macroscopic, and microscopic changes. These changes were evidence of secondary cartilage degeneration and abnormal bone remodeling with inefficient repair and minimal inflammation processes, all of which are similar to the naturally occurring disease in the horse [2–5].

Technical modifications of the grooving procedures used in canine [19–21] and ovine [22] models were required here because of the limited access to the dorsal aspect of the MP joint and the narrowed space between P1 and MC3 in horses. In particular, the arthroscopically controlled technique applied here was distinctly different from the canine and ovine blinded grooving technique, and the instrument used was a customized, modified arthroscopic hook probe with a sharpened 2-mm tip, as opposed to the Kirschner wire employed in the other models. These modifications ensured that surgical cartilage damage was limited to the non-calcified cartilage up to the junction with the calcified cartilage, as demonstrated histologically. Limiting the damage to the cartilage was an important aspect of the technique because we wanted to avoid subchondral bone disruption that would trigger release of cartilage repair factors [28] and OC debris, inducing inflammation [15].

Arthroscopic evaluation of the dorsal aspect of the MP joints showed that two individuals had slight lesions already present before grooving, which might have influenced the results. However, these lesions concerned an area dorsal to and away from the groove location, and during postmortem macroscopic evaluation, they were unchanged. Histologically, the area evaluated did not include these lesions because they were centered on the groove area and the palmar aspect of the condyle. In future extensions of this pilot study, the presence of an existing lesion might be an exclusion criterion.

Clinical lameness evaluation using the American Association of Equine Practitioners’ lameness scale was applied to ensure that horses were comfortable during the study. The absence of blinded clinical lameness evaluation, its subjectivity, and the lack of clinical sensitivity of this type of evaluation were balanced by the objective analysis with an inertial sensor system. Despite an individual disparity in lameness, a significant and consistent variation in forelimb movement asymmetry between the groove and sham-operated limbs was measured with the inertial sensor system. This variation showed a biphasic pattern with an initial increase in groove limb movement asymmetry in the first 6 weeks followed by a decrease in asymmetry. These observations might suggest that lameness could be related to the surgical trauma then to secondary degenerative changes, but no intermediate evaluation was performed to confirm this assumption. The groove model displayed a consistent measurable asymmetry of movement compared to the OC model [14], which does not measure differences using kinematic gait analysis.

Osteophytes were the major visible radiographic signs present throughout the joint. Their presence seems to be species-related because no osteophyte formation has been reported for the original canine groove model [19–21] and osteophytes have been visible only on micro-CT in the ovine groove model [22]. The severity of osteophyte formation is model-dependent in equine OA and seems to be related to the type of cartilage damage [8, 14] and the type of joint [14, 15]. Although JSW is an important indicator of OA in human knees [29], measured JSW was not significantly different in this study. The presence of osteophytes was the major radiographic change visible and was more heavily weighted than synovial effusion, subchondral bone sclerosis, subchondral bone lysis, and JSW in our grading scheme. Histology was not used to evaluate osteophyte formation because we considered radiology sensitive enough for this parameter.

Macroscopic evaluation of the non-surgically damaged area revealed the presence of OA with numerous wear lines, important erosions especially localized on the dorso-proximal aspect of MC3, and slight palmar arthrosis. These changes are observed in naturally occurring OA [6]. In comparison, histologic evaluation performed on slices taken in the dorso-palmar direction did not support these macroscopic findings; the slices were parallel to the wear line and centered over the groove area, away from secondary erosions. Interestingly, although major changes were observed in the anatomical contact region within the groove area, similar lesions were also observed in other areas of the joint. These changes were not expected because the canine and ovine groove models show localized microscopic changes only around a groove. This equine groove model seemed to display more severe macroscopic changes than either the impact injury model [8] or the non-terminal OC model [14], but comparison is difficult because lesions in the OC model were evaluated arthroscopically.

A standardized histological and histochemical OARSI grading [27] system was used for comparing different models equally. Using standard histologic OARSI grading [27], scores would have been overestimated by the vertical surgical grooves. For example, because of the surgical defect, chondrocyte necrosis, cluster, fibrillation/fissuring, focal cell loss, and stain uptake scores would have been 4/4 for all groove joints, making it difficult to highlight specific microscopic features of the model. In addition, because the surgically induced grooves were limited to the non-calcified cartilage and secondary changes seem to differ depending on the layer of the cartilage, it was interesting to evaluate the subchondral bone and the cartilage with non-calcified and calcified sublayers separately. Additional features were added to allow evaluation of primary surgical grooves (vertical groove) and secondary changes in cartilage (horizontal cracking, detachment of non-calcified cartilage). Also, the OARSI guidelines for microscopic osteochondral lesion grading apply criteria involving fragmentation, debris, fibrin, fissure, comminuted fracture, collapse of osteochondral tissue, subchondral bone remodeling, and subchondral bone splitting, all of which are more adapted for advanced damage to the subchondral bone. In the present groove model, most of these criteria would have a score close to 0/4, and subtle specific microscopic features like osteoblastic margination, edema, congestion, and subacute inflammation would not have been highlighted. Furthermore, P1 and PSB were not assessed histologically in our study because, based on the canine and ovine groove models, we did not expect to have lesions at a distance from the grooves.

Histologic examination of the surgically damaged cartilage and subjacent tissues confirmed similar marked vertical grooves limited to the non-calcified cartilage layer in all joints. In the grooved areas, horizontal cracking, bare calcified cartilage, focal detachments of non-calcified cartilage, and cartilage fragments were considered to have developed secondary to altering the cartilage and were at a distance from those made during arthroscopy.

In the vicinity of these major findings, the non-calcified cartilage chondrocyte displayed necrosis (i.e., focal cell loss evidenced by chondrocyte-free areas and empty chondrocyte lacunae), chondrocyte clustering (early regeneration or intrinsic repair), and cartilage matrix softening attributed to loss of fibrillary and interfibrillary contents with some edema. The bare calcified cartilage also displayed necrosis and fissuring, and some fissures extended into the subjacent subchondral bone, with rare openings into the cancellous bone cavities. At the bottom of a few grooves, a thin superficial fibrosis originating from the underlying channeled calcified cartilage and subchondral bone (vascular channeling) was interpreted as early healing.

In the underlying subchondral bone, osteoblastic margination, in association with congestion, edema, fibrin deposition, subacute inflammation, fibrosis, and micro-osteophytosis was indicative of a reactive osteogenetic and inflammatory diffuse reaction. Cartilage vertical grooving, horizontal cracking, and detachment of non-calcified cartilage were worse on the lateral than the medial condyles. These differences might have resulted in differences in OA induction, anatomical constraints, or the worsening effect of repeated biomechanical trauma on the lateral condyle during exercise. On the palmar aspect of the MC3 condyles, very few fissures were noted, with associated non-calcified cartilage necrosis, clusters, and matrix softening. Subchondral bone edema and osteoblastic margination were frequently noted and were considered local extensions of findings of the dorsal aspect of the MC3 condyle.

None of the bone and cartilage biomarkers of turnover were significantly different between groups. Other OA models have shown a significant elevation in CS846, CPII for the OC fragment-plus-exercise model in the mid-carpal joint [30], and in COMP for the impact injury model of the palmar aspect of the MC3 [8]. These differences might be the result of differences in experimental conditions such as damage to the subchondral bone layer, the intensity of exercise, and the study duration. In the present study, the increase in synovial fluid TP without PGE_2_ or leukocyte modification in groove joints implies a release of proteins that have yet to be identified. Earlier markers for joint degeneration such as CTX-II [31], proteoglycans, and glycosaminoglycans [19] would have been interesting to evaluate in synovial fluid and tissue. The significant presence of chondrocyte in the groove joints on day 70 was the first sign of joint degeneration in the synovial fluid. The absence of significant differences in synovial fluid neutrophil between groups suggest that cartilage grooving did not produce significant inflammation. However, neutrophils were slightly present in both groups with a trend towards a common decrease between day 3 and 70, suggesting a slight inflammation induced by the arthroscopic procedure.

The groove model is a traumatic injury model of OA that differs from the OC and impact injury models in terms of type of injury and the degree of secondary deterioration. The groove model allows characterization of primary non-calcified cartilage lesions followed by secondary deterioration of non-calcified cartilage, calcified cartilage, and subchondral bone. The OC model, on the other hand, allows for primary subchondral and cartilage lesions followed by secondary cartilage changes [14], and the impact injury model generates slight cartilage changes secondary to superficial cartilage impact [8].

Unlike other equine MP joint OA model, the groove model is not intended to mimic a specific event of the natural disease. Thus, the cartilage grooves created for this study were not intended to mimic wear lines but rather to serve as standardized non-calcified cartilage defects created to generate predictable secondary degenerative changes. The groove model offers several advantages over the other options to assess the beneficial effects of therapy in OA, including more severe radiographic, macroscopic, and histologic changes in the entire joint; a shorter evaluation period; a consistently measurable asymmetry of movement; the ability to follow osteophyte progression using imaging methods; and the presence of bio-mechanical constraint. In addition, it offers physiopathological perspectives such as the study of the mechanism of chondrocyte necrosis and early subchondral bone anomalies.

This pilot study using six horses demonstrates that an arthroscopic method of non-calcified cartilage grooving combined with exercise results in degenerative changes with significant clinical, radiographic, macroscopic, and histological features, inefficient repair, and little inflammatory response.

## Supporting Information

S1 Abstract Proceeding of the European College of Veterinary Surgeons. Annual scientific meeting, July 4–6, 2013, Rome, Italy. Title: Experimental model of metacarpo-phalangeal degenerative joint disease in adult horses: an equine groove model.
(PDF)

S1 ARRIVE Guidelines Checklist The authors followed the ARRIVE (Animal Research: Reporting of *In Vivo* Experiments) guidelines. (PDF)
