# Supplementary material for: Development of an Equine Groove Model to Induce Metacarpophalangeal Osteoarthritis: A Pilot Study on 6 Horses
Source: PLoS One. 2015 Feb 13;10(2):e0115089. doi: 10.1371/journal.pone.0115089 (PMC4332493; doi:10.1371/journal.pone.0115089)
Supplement: S1 Abstract — Annual scientific meeting, July 4–6, 2013, Rome, Italy. Title: Experimental model of metacarpo-phalangeal degenerative joint disease in adult horses: an equine groove model. (PDF) [file pone.0115089.s001.pdf]

Close this window to return to IVIS
http://www.ivis.org

Proceeding of the European College of Veterinary Surgeons Annual Scientific Meeting ECVS

July 4–6, 2013
Rome, Italy

Next Meeting:

July 3-5, 2014 – Copenhagen, Denmark

Reprinted in the IVIS website with the permission of the
European College of Veterinary Surgeons

Reprinted in IVIS with the permission of ECVS Close this window to return to IVIS

Experimental model of metacarpo-phalageal degenerative joint disease in adult horses: an equine groove model

Maninchedda UM[1], Lepage OML[1], Steinberg RS[2], Hilairet SH[2], Cortez PC[2], Remandet BR[2], Meot FM[2], Penarier GP[2], Segard ES[1], Gangl MG*[1]*

[1]University of Lyon; VetAgro Sup, Veterinary Campus of Lyon, GREMERES-ICE Lyon Equine Research Centre, Marcy l'Etoile, France, [2]Sanofi-aventis Recherche, Montpellier, France.

Introduction

There is no equine model of metacarpophalangeal (MP) degenerative joint disease (DJD) that enables the assessment of progression and treatment of this condition. In order to create an equine MP DJD model with similar characteristics to the natural disease, a surgical procedure similar to the canine groove model was developed.

Material and methods

Standardized cartilage "grooves" were created on the lateral and medial weight-bearing areas of randomized metacarpal condyles of six healthy French Standardbreds using an arthroscopic technique. The other limb was used as a sham-control. After surgery and 10 weeks of controlled exercice, horses were evaluated by clinical lameness examination with an inertial sensor system, with fetlock synovial fluid biomarker analysis, and with semi-quantitative grading of radiography, low-field MRI, cytology, histology, and post-mortem examination. Comparative descriptive statistics between control and treated joints were performed (significance set at $p<0.05$). Correlations were evaluated using scatter plot. Pearson's coefficient of correlation was considered different from 0 if $p< 0.05$.

Results

The grooving procedure under arthroscopic guidance produced highly reproducible cartilage lesions, as demonstrated histologically by the presence of vertical grooves, horizontal cracking, eburnations, and cartilage fragments. After 10 weeks, cartilage grooving in association with controlled exercise triggered severe degenerative changes in the entire joint, with ethically acceptable lameness (1-3/5 AAEP scale). Compared to control joints, treated joints demonstrated major radiographic DJD changes (median 11.5 +/- 1.5, p=0.0011) including synovial effusion, osteophytes, and subchondral bone sclerosis. Severe macroscopic post-mortem DJD lesions (median 5+-1, p=0.0143) were confirmed by wear lines, erosions, palmar cartilage degradation, and bone remodeling on joint margins. Pearson's coefficient of correlation demonstrated significant correlation (p=0.0251) between post-mortem and radiographic scores. Histological evidence of altered cartilage morphology and cartilage degeneration were demonstrated by eburnations, cracks, chondrocyte necrosis and cartilage matrix softening. Evidence of minimal cartilage repair and early bone scarring was illustrated by chondrocyte clusters, fibrosis, calcified channels, vascular channeling, osteoblastic margination and vascular congestion.

Discussion / Conclusion

These findings demonstrated that the equine groove model induced highly reproducible experimental MP DJD with inefficient repair and little inflammatory response as shown by a mild increase in neutrophils and total protein (p=0.005) and no significant difference in PGE2 levels (p=0.0736) between treated and control joints.

Proceedings of the European College of Veterinary Surgeons Annual Scientific Meeting, 2013 - Rome, Italy
